# Implementation of an open chemistry knowledge base with a Semantic Wiki

**DOI:** 10.1186/s13321-025-01037-w

**Published:** 2025-07-06

**Authors:** Charlotte Neidiger, Tarek Saier, Kai Kühn, Victor Larignon, Michael Färber, Claudia Bizzarri, Helena Šimek Tosino, Laura Holzhauer, Michael Erdmann, An Nguyen, Dean Harvey, Pierre Tremouilhac, Claudia Kramer, Daniel Hansch, Fabian Schönle, Jana Alpin, Maximilian Hartmann, Jérome Wagner, Nicole Jung, Stefan Bräse

**Affiliations:** 1 KIT-Bibliothek (BIB), Karlsruhe Institute of Technology, Kaiserstraße 12, 76131 Karlsruhe, Germany; 2 Institute AIFB, Karlsruhe Institute of Technology, Kaiserstraße 12, 76131 Karlsruhe, Germany; 3 DIQA Projektmanagement GmbH, Amalienbadstrasse 41 (Bau 52), 76227 Karlsruhe, Germany; 4 Institute of Biological and Chemical Systems, Functional Molecular Systems (IBCS), Karlsruhe Institute of Technology, Kaiserstraße 12, 76131 Karlsruhe, Germany; 5 Institute of Organic Chemistry, Karlsruhe Institute of Technology, Kaiserstraße 12, 76131 Karlsruhe, Germany; 6 Hue city, Vietnam; 7 Karlsruhe Nano Micro Facility (KNMFi), Kaiserstraße 12, 76131 Karlsruhe, Germany; 8 Department of Chemical Science and Technologies, University of Rome “Tor Vergata”, Via Della Ricerca Scientifica, 00133 Rome, Italy

**Keywords:** Open science, MediaWiki, Semantic web, FAIR data, Databases

## Abstract

In this work, a concept for an open chemistry knowledge base was developed to integrate chemical research results into a collaboratively usable platform. To achieve this, we enhanced Semantic MediaWiki (SMW) to support the collection and structured summary of chemical data contained in publications. We implemented tools for capturing chemical structures in machine-readable formats and designed data forms along with a data model to ensure standardized input and organization of research results. These enhancements allow for effective data comparison and contextual analysis within an expandable Wiki environment. The use of the platform was specifically demonstrated by organizing and comparing research in the area of “CO_2_ reduction in homogeneous photocatalytic systems,” showcasing its potential to significantly enhance the collaborative collection of research outcomes.

**Scientific contribution**

This work shows ways to collaboratively collect and manage subject-specific knowledge in the domain of chemistry via an open database. By integrating cheminformatic tools into Semantic Mediawiki, an established technology for building knowledge databases is made systematically usable for the chemical community. The integration of chemistry-specific workflows and forms allows the mapping of data from current research with links to the original sources. This work is intended to show how gaps in the information system of scientists can be closed without having to use commercial systems.

**Supplementary Information:**

The online version contains supplementary material available at 10.1186/s13321-025-01037-w.

## Background

**Fig. 1:**
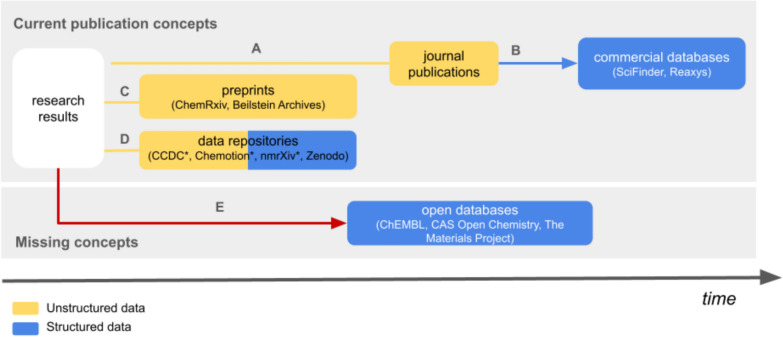
Schematic description of the current process of sharing unstructured and structured information in the domain of chemistry by the publication of manuscripts in journal publications (**A**), submission of preprints (**C**) and the disclosure of research data in repositories (**D**). After the publication in journals, commercial databases are built via path (**B**) while public open databases with information on scientific investigations and their results **E** are rare for many areas of research (such as in chemistry). Examples with assigned “*” contain structured data

The provision and preparation of information and knowledge is a major challenge in science. A coherent and focused presentation of scientific facts that is accessible to all scientists is a complex goal that should include solutions for providing overviews, individual scientific articles, metadata and data. The prevalent method for disseminating new data, information, and findings within most disciplines involves publication through traditional publishers (Fig. 1A). This process has the advantage of vetting information through established and recognized processes such as peer reviewing in order to separate trustworthy, high-quality scientific information from that which is not [1, 2].The problems of the current process of publishing results via mostly journal publications are (1) the poorly structured, and mostly neither standardized nor machine-readable presentation of results and thus the more difficult subsequent use of the knowledge contained in the publications, (2) the, in many cases, not yet open direct provision of all content due to low dissemination of gold open access procedures, and (3) the mapping of the respective status quo, which is mostly already outdated by the time publications are disclosed. For some years now, journal publications have been supplemented in part by data publications (Fig. 1D), which increase the transparency of science and—if data provision is carried out in accordance with standards established in the community in subject repositories—enable faster and better structured availability of data. The structured, standardized, open provision of research data in repositories can be an essential pillar for making information available in the future [3, 4] For the scientific contributions themselves, pre-print servers (Fig. 1C) can be a solution for rapid publication, so that results are open and quickly accessible. To date, however, there are few processes that support the systematic provision of structured information through the currently established publication processes. Structured information can be obtained in some disciplines in the form of databases, but some databases require users to pay for a license. Typically, the content of the databases is collected by commercial providers reusing the information of scientific publications by extracting, curating, and processing the data contained in the publications for structured presentation (Fig. 1B). The databases obtained by processing published data and information are of great importance for scientific work, especially in chemistry where the databases SciFinder [5] and Reaxys [6]. They form the information backbone for searching scientific information. For chemical reactions, known chemical substances, and properties of substances, the databases SciFinder [5] and Reaxys [6] are an important part for literature and information search. However, the use of the commercial systems is associated with high costs and the provision of information takes place with a time delay, as the systematic investigation and re-use of non-structured information from the literature takes time, even for commercial providers. Efficient processes to gain structured information need either laborious manual extraction or software-supported analysis [7–9]. Other disadvantages of providing structured information via commercial databases are the low influence of the chemical community on the presentation and curation of the information and the lack of means to efficiently provide overviews about subject-specific content. While formats such as Wikipedia can be used to provide and query general knowledge, there are no comparable platforms for current research content, and overviews for current research topics have to be generated manually from scratch in the form of review articles. However, these are quickly outdated and can only provide a static insight into the prepared topic. In the long run, AI based on large language models (LLMs) could probably serve as a valuable extension to gain state of the art insights and customized overviews on research topics. Large Language Models (LLMs) have shown robust capabilities for knowledge extraction across various domains [10] and may be used to fill the gap between unstructured information and users in chemisry [11–14]. However, their performance on the current state of the research is not yet comparable with data collections that are curated by scientists [15–17]. In summary, we are currently in a situation where science could greatly benefit from the availability of large datasets, as machine learning methods could accelerate knowledge gain in many disciplines based on these data. Particularly in the field of chemistry, the combination of data and the use of cheminformatics tools could lead to significant advancements [18–20]. Unfortunately, there are still only a few open datasets and platforms (e.g., PubChem [21], ChEMBL [20], the Materials Project [22]), and the lack of available data limits the use and application of AI. While the larger part of the scientific chemistry community still relies on the information provided by a few commercial databases, the advantages of opportunities of community-driven and -curated platforms are currently often not taken.

## Results

### General design and components

In order to find a solution for the current situation, a pilot project has been launched to demonstrate a way to combine currently established mechanisms of publishing scientific data with community-based processes and the possibility to present information in a structured way. The aim is to develop an open platform for collaborative work to make research results available in the field of chemistry. The platform needs to be suitable to include community-driven content generation and curation to ensure a high quality of the content without dependencies on commercial systems. It further needs to support options to include automatic checks and curation to support the identification and correction of errors.

The pilot project uses the example of photocatalytic CO_2_ reduction to demonstrate how chemical content can be incorporated into knowledge platforms and to show ways in which faster access to and interpretation of structured information can be achieved in the future. The example of chemistry was used to show how the creation of such a platform for the alternative provision of information can succeed as an implementation with complex, discipline specific requirements. The main criteria for the design of the project and the selection of the tools were (1) public accessibility to information, (2) the possibility of active contribution of content by the community, (3) the possibility of continuous further development of the content, (4) the possibility of semantic processing of the content, (5) the open provision of the source code and available documentation, and (6) the adaptability of the technology by the integration of an active developer community. The setup of the project in the field of chemistry resulted in further requirements for the data parsing components to be included and the workflows that must be established within the knowledge platform in order to achieve its acceptance by scientists. In chemistry, it is particularly important in almost all sub-disciplines to support the drawing, representation and processing of chemical structures. For this purpose, drawing tools for molecular structures have to be integrated, which allow easy representation of subject-specific standards in a drawing editor and their storage in a human- and machine-readable way. The human-readable representation is achieved by generating an image that can be embedded in web pages or documents, while the machine-readable representation can be obtained in the form of various text-based exchange formats and identifiers that allow matching with other databases and structuring of content within a knowledge platform.

### Technical implementation

Various collaborative platforms such as Wikibase were considered for the technical implementation. Core aspects for the selection were the possibility to use the platform without special technical knowledge, to store entered data in a machine-processable way and to be able to enter chemical formulas. Semantic MediaWiki (SMW) [23] (especially in combination with the extension “Page Forms” [24]) was chosen because it is based on the user-friendly and widely used MediaWiki software which includes functions such as versioning of the contributions for driving the wiki’s content by the community. Besides the option to store information in an unstructured way which is needed to provide scientific information in textual form, SMW offers additional functions that allow data entered in human-readable format to be stored in a structured and machine-readable way (for limitations of SMW see SI Sect. 11). The beneficial combination of features for structured and unstructured information as given in SMW was not found in other related wiki developments such as Wikidata. Another benefit of SMW is in particular that a semantic model of the data can be defined initially and corresponding input forms can be created. These allow domain experts from chemistry to enter structured information even without special knowledge of the wiki software or the underlying technologies from the Semantic Web area. Information entered in this way is available in the system in a structured and semantically described form, enabling fine-grained search queries and the integration with external data sources. Additional extensions to Semantic MediaWiki provide visualization, import, and export capabilities, which promote knowledge sharing and collaboration with domain experts outside of chemistry as well as external technical systems.

### Structure of the content

**Fig. 2:**
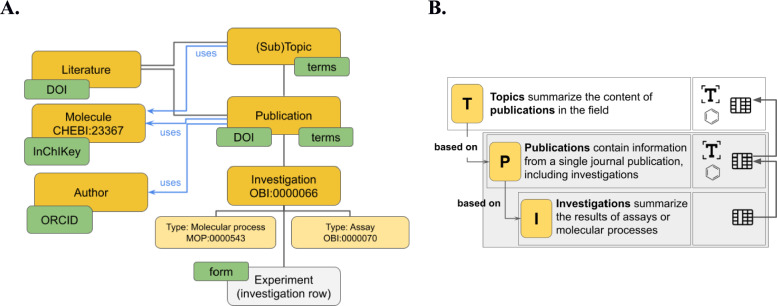
**A** Schematic representation of the conceptual data model of the CKB project: Yellow boxes represent the entities implemented as individual pages in the SMW, green boxes indicate mechanisms to formalize and standardize contents of the pages. **B** Schematic representation of *Topics* (T), *Publications* (P), and *Investigations* (I) which are the main content pages within the Chemistry Knowledge Base (CKB). *Topic* pages are based on *Publication* pages which are based on *Investigation* pages. Table content from the *Investigations* is used to enrich tables in *Publications* and *Topics*. *Investigations* always belong to *Publications* and cannot be generated without

Our developments resulted in the establishment of the MediaWiki-based Chemistry Knowledge Base (CKB), which is based on known practices in the community to provide reviews based on well-documented scientific results. The CKB content includes *Topics*, *Publications* and *Investigations* as essential components of the main Wiki pages. The additional pages *Molecules* and *Literature* allow the itemization of chemical structures and literature articles or datasets—both needed to describe the content of *Topics*, *Publications* and *Investigations* (Fig. 2). *Topic* pages summarize information that allows an overview of the results in a scientific research field, containing text, tables and molecular drawings. It is important that the presented information is supported by the citation of literature or individual references to *Publication* pages within the Wiki. The creation of *Publication* pages allows for the itemization of content in *Topic* Pages. As such, they deal with the essential content of a scientific journal publication or else a data publication. The *Publication* page provides the ability to map the scientific content of a publication using text, molecular drawings, and tabular summaries of data. It can refer to a scientific article (here: *Literature*) or other information by linking an existing DOI of the scientific article or information to the corresponding SMW page. If no DOI can be assigned, the Publication page is identified by its title only. The tabular summaries within a *Publication* page play a special role, as their content is created via the so-called *Investigation* page. The *Investigation* page displays the values obtained from a scientific experiment in a standardized form. Standardized templates are offered for each of the supported investigation types such as *Molecular Process* or *Assay* (Fig. 2, yellow panels), so that a comparative input and display of the respective experiments is possible both on the *Publication* pages and later on the *Topic* pages. This is possible because the *Investigations* can be queried for specific properties across *Publications* using Semantic MediaWiki Queries via “Investigation Links”. By using ontologies, concepts in a data model are assigned to terms in a controlled vocabulary. This has the benefit that knowledge encapsulated by the data is represented in a way that is structured and coherent across systems. We specifically utilize the external ontologies OBI, ChEBI, and MOP to define classes in our data model and integrate them by assigning a special property *imported from* which is provided by SMW*.*^1^ We reused existing concepts whereever known and only added missing parts to the model required for the establishment of the CKB (SI, Sect. 2 and 3). CKB extends SMW with additional means for linking to standardized, external data sources (Fig. 2, green panels). DOIs allow the identification of published elements such as journal articles (embedded in the form of *Literature* or *Publication* pages). A special routine allows the retrieval of further information on DOI-linked items from Crossref (see supporting information). *Molecules* are described uniquely by the generation of chemical identifiers such as InChIKeys and the standardization of *Investigations* is achieved by providing forms for every *Experiment* that is contributed to the *Investigation* page. *Authors* are identified and linked to the relevant articles in the CKB via their ORCID. The *Topic* and *Publication* pages of the Wiki support the assignment of the content to terms from ontologies. This allows the semantic enrichment of the included information for search and further data representation purposes (Fig. 2).

### Topic and subtopic pages

**Fig. 3:**
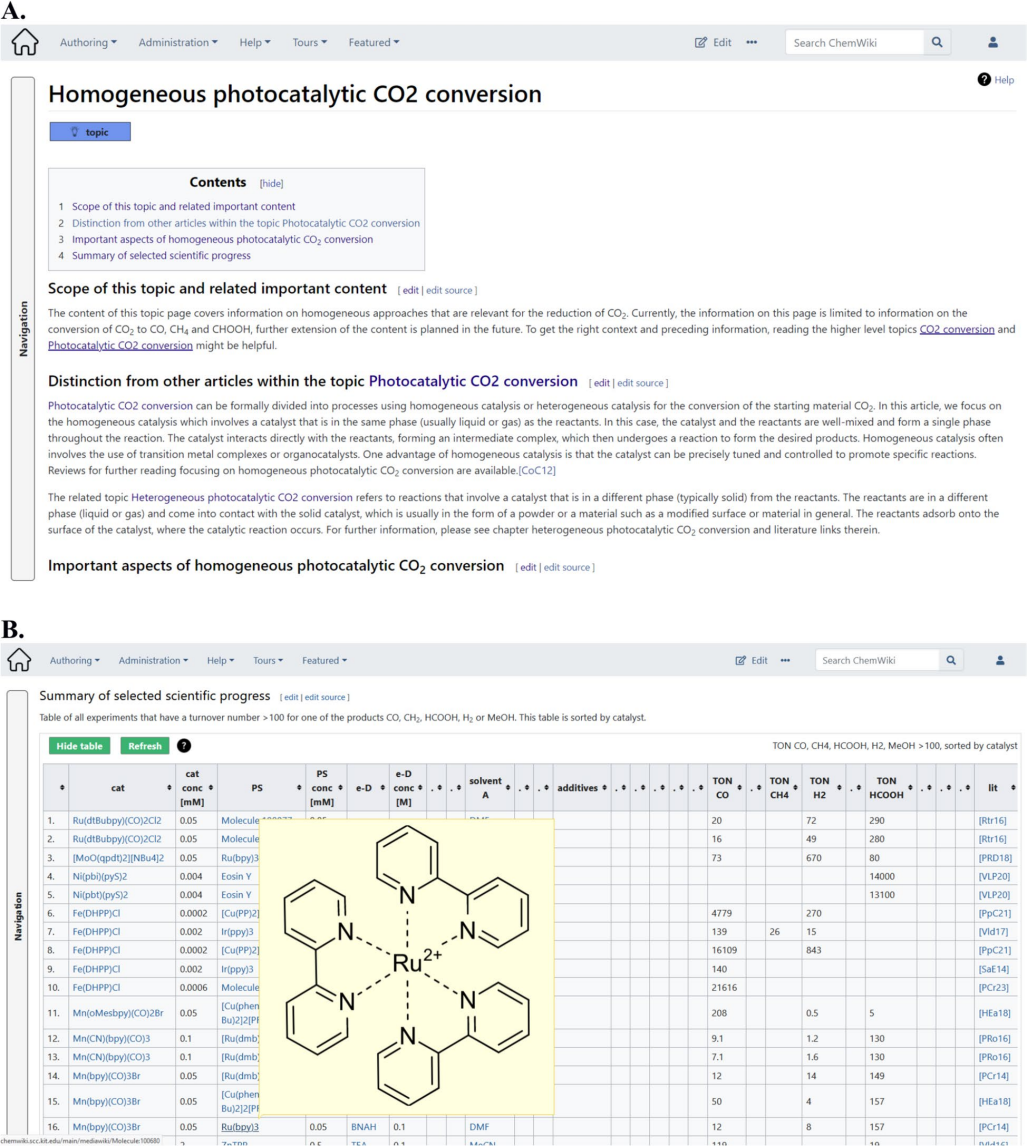
Screenshot of a typical *Topic* page in the CKB Wiki. **A** The first part of the page consists of (1) a summary of the content, and (2) a textual description of the relevant field which can be divided into several sections. **B** Tables complete the overview about the recent state of the developments and offer the option to compare results based on the achieved experimental results. Each molecule, e.g. a catalyst or sensitizer, is shown with its full structure upon hovering over the name or number of the molecule in the table

(Sub)*Topic* pages give an overview of specific scientific fields. They aim to summarize the current state of research in text, tables and by presenting relevant molecules and chemical compounds (Fig. 3A). The latter are rendered as images to facilitate the comprehension (see “Insertion, processing and presentation of chemical structures“, Fig. 5, and SI chapter 1 and 8). *Topic* pages can be supplemented by subtopic pages and those again by even more specific subtopic pages. This allows a presentation of information in multiple layers of granularity. Using the interconnections of links to other pages in the Wiki, each page focuses on one specific topic and has references to the pages related to it, such as parallel topics or broader/narrower overviews. The (Sub)*Topic* pages usually consist of a content area, textual descriptions that are relevant for the scientific field and the understanding of the topic, molecules (in different representations such as images) that allow the chemical understanding, and tables that summarize the current state of the scientific progress. The tables embedded to a (Sub)*Topi*c page play a special role in the CKB Wiki as they consist of table entries that are fetched from the *Publication* pages related to this (Sub) *Topic* (Fig. 3B). According to the implementation of the linkage rules depicted in Fig. 2A, each line in a (Sub)*Topic* table corresponds to a line of a *Publication* page table (i.e. coming from an *Investigation* that is part of the *Publication*). The tables are therefore not entered in the (Sub)*Topic* page, they are automatically fetched (queried) from other pages in CKB. (Sub)*Topic* pages also include information on the available further (Sub)*Topic* pages which are linked and contain information on related publications and literature. Related publications are considered those scientific articles that are available in the Wiki as *Publication* pages. Literature information includes references to articles that are not part of the Wiki, approving the statements given in the (Sub)Topic pages by additional information available in the form of web-links. *Publication* pages are always referenced with *Literature* pages if the *Publication* refers to results described in a journal article.

### Publication pages

*Publication* pages give a summary of the information that is described in journal articles or datasets. They summarize the main content of publications in the form of text, tables and molecules and are therefore a resource to get information on journal articles even if they are not available under an open access model. *Publication* pages serve also as resources to build the content of the (Sub)*Topic* pages via transclusion as the content of the investigation tables within *Publications* can be used automatically as input for tables in the (Sub)*Topic* pages. *Publication* pages are complemented with metadata and supplemental information, such as the infobox containing bibliographic data (from the automatically generated “literature page”), and molecules involved in the scientific description of the original publication. While the structure of a *Publication* page is usually not fixed and can be designed by the author of the page, *Publication* pages describing certain fields of research should follow guidelines such as that pages about the photochemical reduction of CO_2_ should contain a section with structures of the discussed catalysts, photosensitizers and further chemical components if relevant. Also, at least one investigation should be part of the *Publication*.

### Investigation pages

**Fig. 4:**
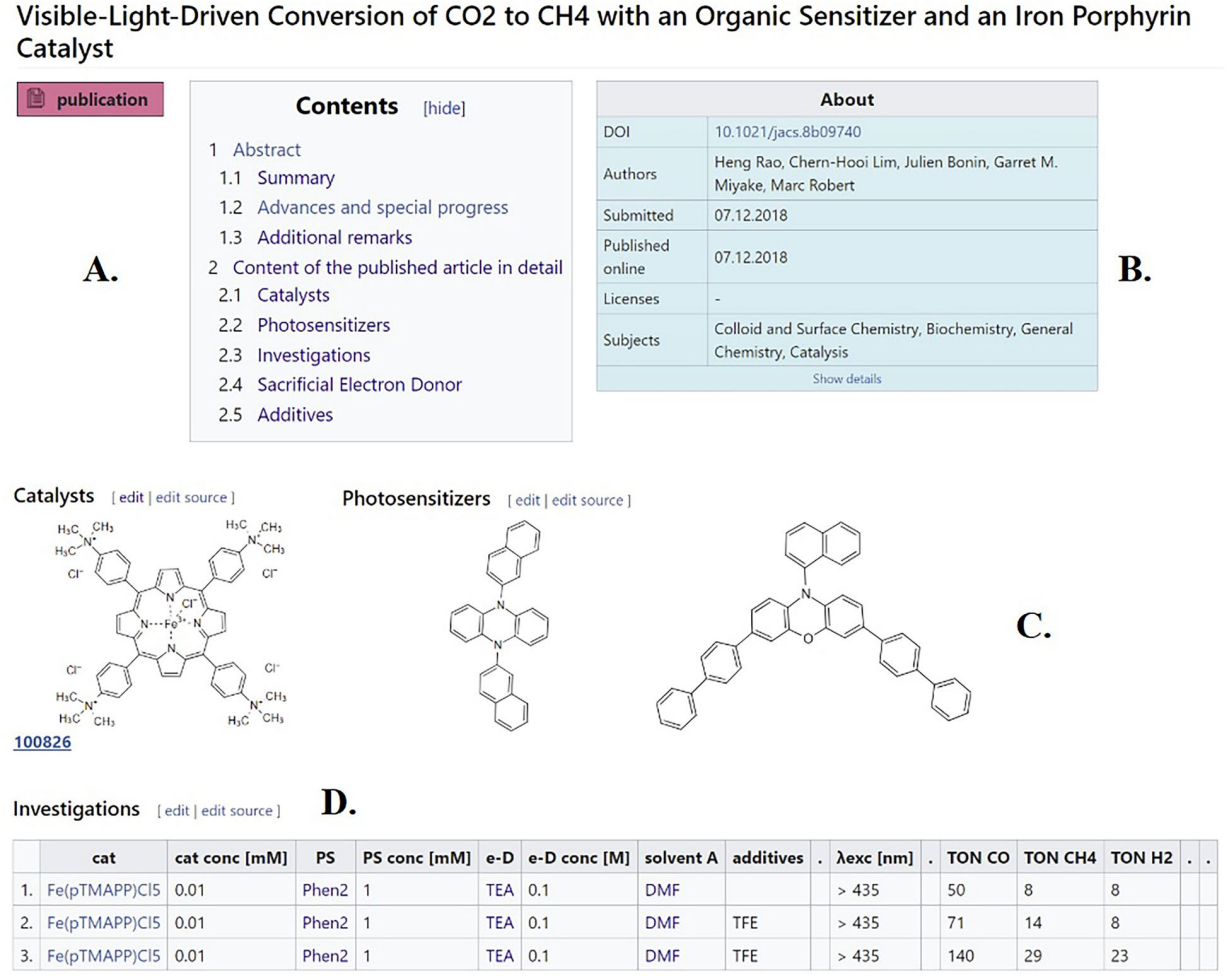
Summary of selected parts of the sample Publication page “Visible-Light-Driven Conversion of CO_2_ to CH_4_ with an Organic Sensitizer and an Iron Porphyrin Catalyst” (original article published by G.M. Miyake, M. Robert et al.) [25]. Amongst other content, *Publication* pages contain **A** the contents of the *Publication* page, **B** an about section giving details on the referenced published article, **C** the structures of the relevant molecules used in the study, and **D** a table summarizing the experiments that were conducted

*Investigation* pages are subpages of *Publication* pages and typically contain the data from experiments of a journal publication. The investigation tables consist of one or multiple *Experiments* (Fig. 4D), equivalent to an individual row in the table. The *Investigation* page is generated by a template. Inserting the investigation template on a publication page opens a form into which the data of an experiment is added. Currently, investigations are classified into two types: molecular processes and assays. Molecular processes are chemical reactions in which a starting material is changed into a product. Assays are physical measurements of a chemical compound. Within those types, specific forms for individual reactions or measurements can be defined according to already standardized representations in journals, ELNs or established practices in the literature. To date, there are two examples in the Wiki, “Photocatalytic reduction of CO_2_” for molecular processes (in productive use) and “cyclic voltammetry” for assays (under development).

### Insertion, processing and presentation of chemical structures

Within the CKB Wiki, molecules are not static illustrations, each molecule is an object of its own, which is stored as its own page, a *Molecule* page. Thus, this page can be referenced in any context and the molecule only needs to be drawn/created once even if it is used multiple times within the Wiki. The description of the molecule is done using standards and the identifiers common in the chemical community, InChI/InChIKey and SMILES which allow the molecule to be uniquely named and referenced (with certain known limitations) [26, 27]. This allows it to recognize the molecule in any representation and to retrieve data from other databases such as PubChem [28]. *Molecule* pages are assembled by a template to provide information on each molecule including the internal molecule ID as the title, the structural formula, the SMILES, InChI and InChIKey, a table with relevant physical data (retrieved from PubChem [28] or ChemScanner [7]), a link to the PubChem entry if that exists,, and a list of all the pages in the Wiki on which the molecule appears. As the internal molecule ID is difficult to remember and does not provide information about the molecular structure, we additionally introduce a system of abbreviations for molecules to facilitate the identification in tables and improve readability and recognition throughout the Wiki. Due to considerations of practical use and space constraints, these abbreviations aim to be shorter than formal IUPAC naming, while still providing a good idea of the molecules’ structure. The rules after which abbreviations are formed draw from both standardized IUPAC naming guidelines and de facto standards established in the community for specific chemical compounds (e.g. bipyridine ligand = bpy). The simple creation of a molecule and its referencing in various contexts enables the linking of information on different Wiki pages on the basis of the molecule.

**Fig. 5:**
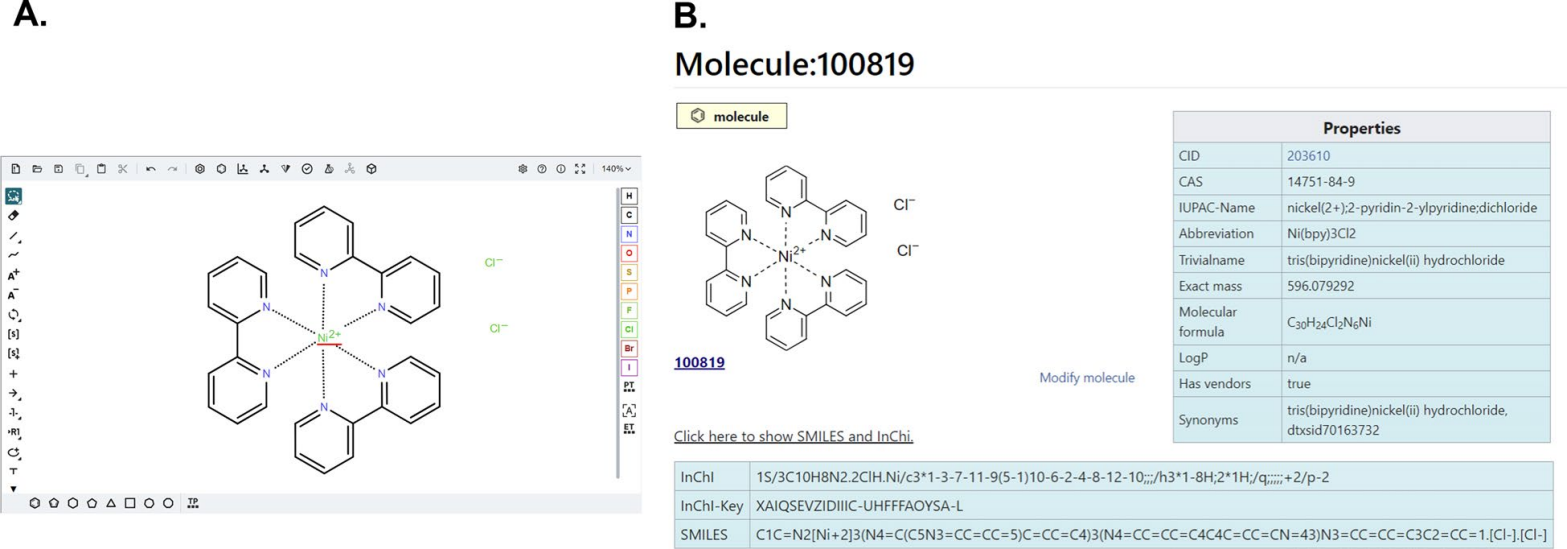
**A** Ketcher editor integrated in MediaWiki’s VisualEditor used to draw chemical structures. **B** Summary of contents describing the molecule on a separate Molecule page in the Wiki. The shown screenshot was adjusted to fit into the available size of Fig. 5

The implementation of *Molecule* pages with unique identification of molecules on different Wiki pages required the embedding and use of the software packages Ketcher [29, 30] and ChemScanner [7] (both using other libraries such as RDKit [31]). The systems are available as open source, but had to be adapted for the integration with MediaWiki. The JS version of Ketcher is used within the Wiki to create molecule drawings and to receive and store the information generated from them. This includes the structure definition in the form of the molfile (MDL MOL) [32], the identifiers InChI/InchiKey and SMILES, as well as the image of the molecular structure, which is important for the users of the Wiki. Ketcher runs fully in the browser but additional adjustment of the rendering of the molecules was externalized into a backend service. We integrated JS-Ketcher into MediaWiki’s VisualEditor so that users can draw diagrams in the context of authoring Wiki pages (Fig. 5A). In addition, the software ChemScanner [7] is needed for various processes such as the parsing of molecular groups with residues that cannot be achieved by Ketcher. The user needs to define the valid residues for a molecular group (by a set of pre-defined abbreviations) in VisualEditor (see supporting information) and ChemScanner transforms them to individual molecules and creates a page for each. ChemScanner also provides information such as molecular formula and exact mass for molecules which are not yet known in the PubChem database.

### Future work

In future enhancements to the CKB, the integration of Large Language Models (LLMs) can play an important role for improving how chemical knowledge is formalized and accessed. One of the primary uses of LLMs in the CKB will be to transform unstructured data from scientific publications into the structured formats used within publication pages, investigations, and descriptions of molecules. To optimize the accuracy and user trust in this automated process, an “assistant” mode, allowing users to review and adjust the extracted values as needed, could leverage the advanced capabilities of LLMs while aligning with the needs and workflows of CKB users. Additionally, enhancements involving a natural language query interface could allow users to interact with the system using everyday language, significantly lowering the barrier to accessing complex data.

## Conclusion

In this work, a concept was developed based on Semantic MediaWiki (SMW) that allows the integration of results from chemical research in a semantically usable form within a Wiki. The results are available in the form of the Chemistry Knowledge Base (CKB) which was exemplarily enriched with data from the field of photochemical homogeneous CO_2_-reduction. One main benefit of the CKB is the successful integration of chemical structures in a machine-readable way by implementation of a chemical structure editor. The storage and representation of structured and standardized information is gained by the provision of forms to enter scientific data and the generation of unique identifiers for molecules. Additionally, semantic models were integrated to facilitate the semantic capture of selected content. The CKB can be extended by the community content-wise as an alternative strategy to the use of commercial databases. We could show how the currently established processes for the publication of scientific results could be used as a starting point to collect relevant information in the field and to summarize the current state of the art in an easy and partly automated way. Due to the manifold benefits of the CKB already in its current state, further improvements should establish the CKB as a tool for open science in the long run. The full semantic description of the contents, the representation and implementation of chemical reactions, and the extension to other fields in chemical science are next aims of the project group.

## Supplementary Information

Supplementary material 1.

## Data Availability

The implementation as described is available under http://chemwiki.scc.kit.edu/main/mediawiki/Main_Page. The code of the developments is available on github: https://github.com/ComPlat/ChemistryKnowledgeBase. The documentation of adaptations and extensions to MediaWiki are described on github https://github.com/ComPlat/ChemistryKnowledgeBase/blob/main/doc/ChemExtension.md. For an installation of the CKB, the dependencies on the three services need to be considered: (1) an R-group service which is used from the software ChemScanner (https://github.com/ComPlat/chem_scanner) and enables molecules from collections with R-groups, (2) a rendering service which renders molecules from molfiles and is based on Ketcher, and (3) the terminology service of TIB which is used for the tagging of terms to taxonomies.

## Abbreviations

CKB: Chemistry knowledge base
ChEBI: Chemical Entities of Biological Interest
DB: Database
DOI: Digital object identifier
ELN: Electronic laboratory notebook
LLMs: Large language models
OBI: Ontology for Biomedical Investigations
InChI: International Chemical Identifier
IUPAC: International Union of Pure and Applied Chemistry
ID: Identification
MOP: Molecular Process Ontology
SMW: Semantic MediaWiki
SMILES: Simplified molecular input line entry specification

## Footnotes

1 See https://www.semantic-mediawiki.org/wiki/Help:Special_property_Imported_from.
