# Supplementary material for: Implementation of an open chemistry knowledge base with a Semantic Wiki
Source: J Cheminform. 2025 Jul 6;17:99. doi: 10.1186/s13321-025-01037-w (PMC12232763; doi:10.1186/s13321-025-01037-w)
Supplement: Supplementary file 1 — Supplementary material 1. [file 13321_2025_1037_MOESM1_ESM.pdf]

Supporting Information

Exemplary adaptation of Semantic MediaWiki to serve as an Open Chemistry Knowledge Base

Charlotte Neidiger[a], Tarek Saier[b], Kai Kühn[c], Victor Larignon[d], Michael Färber[b], Claudia Bizzarri[e,h], Helena Simek[d], Laura Holzhauer[d], Michael Erdmann[c], An Nguyen[f], Dean Harvey[d], Pierre Tremouilhac[d], Claudia Kramer[a], Daniel Hansch[c], Fabian Schönle[d], Jana Alpin[d], Maximilian Hartmann[d], Jérome Wagner[d], Nicole Jung[d,g], Stefan Bräse*[d,e]*

Content

1. Further information on options to structure the SMW 1
2. Structure of the Chemistry Knowledge Base/ Schema 3
3. Wiki-Schema 4
4. Annotations 7
5. Technical Implementation of Services 7
6. The Literature page 8
7. Workflow supporting the generation of Molecules and Molecule Groups 9
8. Details for the Molecule Page 12
9. Authors page 12
10. Information on Ontologies and Terms used in the CKB Model 13
11. Main limitations and opportunities of the CKB 14
12. Possible further extensions with respect to LLMs 17
13. Software dependencies 19

1. Further information on options to structure the SMW

The extension imposes a particular data model in the wiki which looks as following:

Topics

Topics are authored compilations of publications. They are represented as sub-categories of the pre-defined category "Topic". The hierarchy of topics builds a tree. It is displayed in the navigation on the left side.

Publications

Publications (aka papers) are a member of at least one topic. They are located in the main namespace. Besides text and images, they usually contain molecules and investigations. Terms given within publication pages can be annotated by selecting a suitable term from established ontologies and vocabularies of the online terminology service of TIB (Hannover)[1].

Investigations

Investigations are sets of experiments. An investigation has a certain type and, according to the type, a particular set of fields. Technically, an investigation is a subpage of a publication, and an instantiation of a multi-instance form.

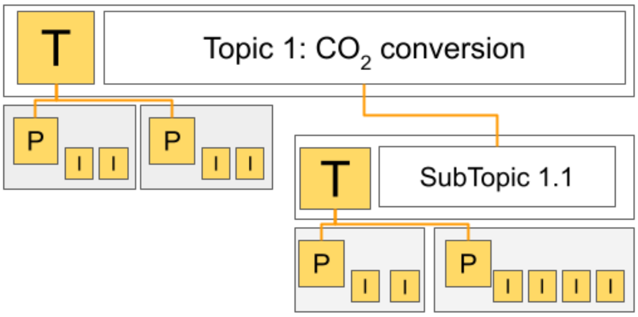

Figure S1. Options to structure the CKB according to topics and subtopics (both built with *Topic* pages), each consisting of *Investigation* page-related *Publication* pages. T = Topic, P = Publication, I = Investigation.

Further chemistry specific content was implemented through molecules and molecule collections:

Molecules and molecule collections

Molecule and molecule collection pages are in a separate namespace "Molecule". Each page there represents one molecule or one collection of molecules (cf. section "Terms") and has a numeric ID which is unique in the wiki. A molecule page contains the structural information as MOL-File and/or SMILES as well as metadata about the molecule like mass or trivialname in a semantic model (For a full list of metadata properties for molecules, see list below in section 2). The InChI-Key of a molecule serves as a global ID for the molecule whereas the numeric page ID is only valid locally in the wiki.

Molecule collections are materialized to separate molecules, each having its own page after that process. These automatically created molecules are linked to the collection page. Such a molecule can be used as any other molecule in the model.

2. Structure of the Chemistry Knowledge Base/ Schema

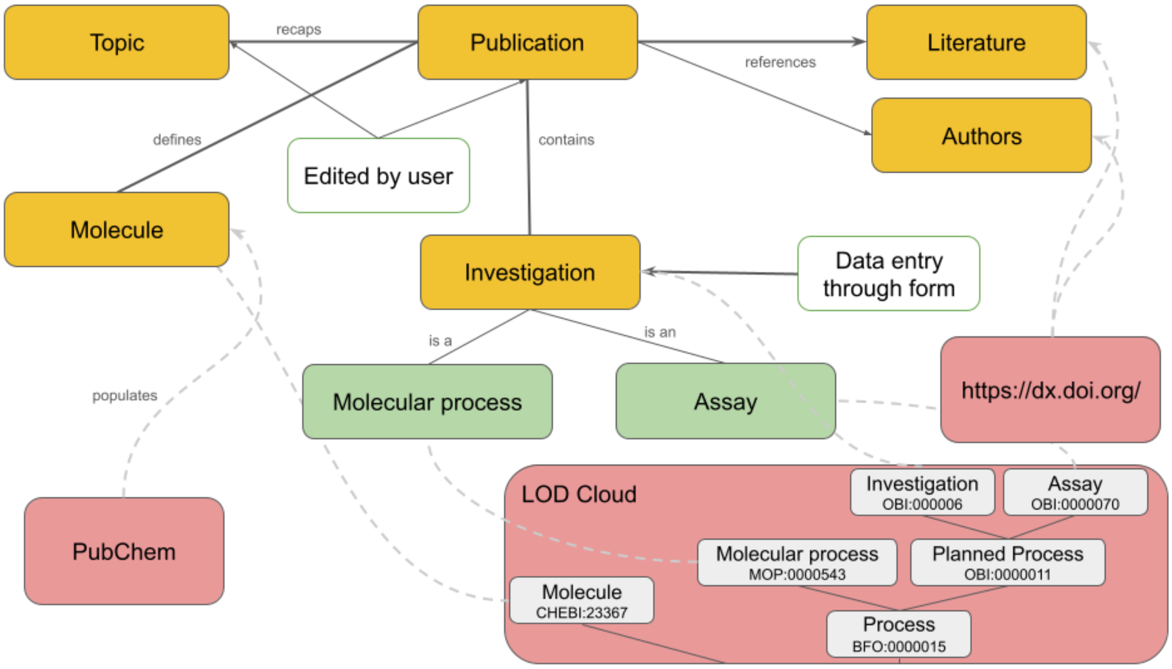

Figure S2. Model, concepts and main components that were implemented in the CKB:

- yellow: top-level concepts within the CKB that are realized in the form of wiki pages;
- red: external services and concepts;
- green: particular instances of investigations;
- white: actions that are required by the user or embedded in the CKB.

3. Wiki-Schema

The wiki basically implements the top-level concepts as instances of its same-named categories. It looks like this:

- Main namespace
  - Main Page as entry point to the wiki.
  - Instances of category "Publication" (empty in new wikis)
  - Instances of category "Investigation" as subpages of publications (empty in new wikis)
- Molecule
  - Instances of category "Molecule" (empty at new wikis)
  - Instances of category "Molecule collection" (empty at new wikis)
- Categories (represent top-level concepts)
  - Molecule
  - Molecule collection
  - Investigation
  - Publication
  - Author
  - Topic
  - Subcategories of Topic (also referred to as "Subtopics")
- Properties (to store data in the semantic model)

- General properties
  - Tag: used for tagging
  - DOI: stores the DOI of a publication
  - Ontology: stores the provenance of a term for tagging
  - OBOID: to identify the term in the ontology
  - Orcid: references an author
  - Publisher: The publisher of a publication
- Investigation related properties
  - BelongsToPublication: Link to the publication page from an investigation
  - BasePageName: Link to a publication page from a subobject in an investigation
  - Unit: Denotes the unit of measurement of a property
  - A set of properties which are related to the type of investigation (e.g. properties relevant to “Photocatalytic CO2 conversion”)
- Molecule-related properties
  - InchIKey: Stores InChI key for a molecule
  - Smiles: Store SMILES string for a molecule
  - Synonym: Stores synonyms for a molecule
  - IUPACName: Stores IUPAC-Name for a molecule
  - CAS: Stores CAS number for a molecule
  - ContainsElement: Stores all different elements used in a molecule
  - MolecularMass: Stores mass of a molecule
  - MolecularFormula: Stores sum-formula for a molecule
  - Abbreviation: Stores abbreviation of a molecule
  - Trivialname: Stores trivialname of a molecule
  - LogP: Stores LogP of a molecule
  - HasVendors: Are there commercial vendors for this substance?

- Templates
  - BaseTemplate: included in all Publication- and Topic-pages
  - Molecule: Included in all molecule pages
  - MoleculeCollection: Included in all molecule collection pages
  - DoiInfo: Used by doiinfobox-parserfunction to render the infobox
  - DisplayMolecule: Display a molecule link

    - Annotation: renders an annotation in the wiki text
    - AnnotationWithOntology: same as above but with a linked ontology
    - Cyclic_voltametry(_experiments): Renders investigation data
    - Photocatalytic_CO2_conversion(_experiments): Renders investigation data
    - PubChem: Renders metadata about molecules
    - Content-related templates to tag content for editors:
        - WrongMolecules
        - MissingMolecule
        - DOINeeded
        - FaultyMolecule
        - NoCategory
        - UnreferencedCategory
- Help
    - Documentation pages to help the user with first steps
- Authors
    - Every instance represents one author. Author pages are automatically created when publications with DOI are added in the wiki.
- Special
    - Literature: Parametrized by DOI. Reads publication metadata from https://www.doi.org/

All static pages are pump-primed pages which are included in all (sub-)wikis. This means that the basic content which is part of the wiki is added automatically to the subwikis (personal work area of a registered user), whenever a subwiki is created. This is the minimum a wiki needs to work properly. This includes pages in the namespaces: Category, Property, Template, Help and Special.

4. Annotations

Besides the top-level concepts which are first-class citizens in the wiki there are annotations which allow tagging words or phrases of a page with terms. They are represented as instances

of the property “Tag” in the semantic model. Its purpose is to facilitate searching by choosing facets and to allow querying groups of publications based on tags.

Tags are preferably chosen from existing ontologies. This is supported by connecting TIB’s terminology service to the wiki. However, annotations can also be chosen completely freely. The system suggests already used annotations to keep up a minimum level of consistency.

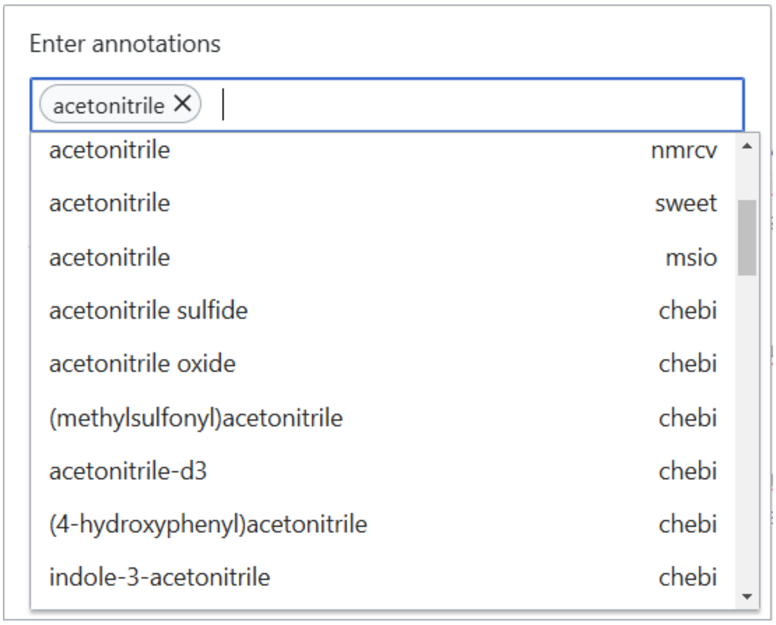

Figure S3. Annotation dialog in Visual-Editor

5. Technical Implementation of Services

ChemExtension[2] needs three external services to work properly. The availability can be checked on Special:CheckServices. They do not use authentication for now.

- *RGroup-Service*
  This service creates concrete molecules from a molecule with R-Group. It turns a series of rows of R-Groups to a set of concrete molecules. The input and output format is MOLFile V3000. The technology used is RDKit (https://https://www.rdkit.org/)
  - Service-URL: http://193.196.36.39
  - Client class: *MoleculeRGroupServiceClientImpl*
  - Base URL configuration: $wgMoleculeRGroupServiceUrl

- *Molecule render service*

  This service renders a molecule in MOLFile V3000 format as SVG-graphic. The technology used is Ketcher as backend-service (https://lifescience.opensource.epam.com/ketcher/index.html)

  - Service-URL: https://dev.ketchersvc.hydrogen.scc.kit.edu
  - Client class: *MoleculeRendererClientImpl*
  - Base URL configuration: $moleculeRendererServiceUrl

- *Terminology service of TIB*

  This service is used to provide a source of chemical terms for the annotation mechanism in the wiki. This facilitates a consistent use of terms and avoids uncontrolled growth.

  - Service-URL: https://service.tib.eu/ts4tib/api
  - Client class: TibClient
  - Base URL configuration: https://service.tib.eu/ts4tib/api

6. The Literature page

Manuscripts that were published in journals are represented in the CKB wiki by publication pages which summarize briefly selected content of the original work. The creation of the publications include the link to a DOI if available - giving the option to retrieve more details from the original publication. A special routine allows the retrieval of further information on DOI-linked items from Crossref. According to the implemented workflow, entering a DOI of a publication while creating a new publication automatically generates a *Literature page*. The metadata provided by Crossref and/or dx.doi.org is shown as part of the *Literature* page and a subset of this information is presented in the infobox at the top of each *Publication page.* This bibliographic metadata ensures the correct referencing of source publications.

Metadata provided with the Literature: ID: DOI; Title; Type; Author: Name, Affiliation (Institution name, Country), ORCID; Submission date; Acceptance/publishing date online/print: min. Year; Publisher: name; Rights/licence; Issue; Volume; Pages; Subjects; Funder.

Metadata provided as About section embedded to the *Publication* page: DOI, Authors, Submitted, Published online, Licenses, Subjects.

7. Workflow supporting the generation of Molecules and Molecule Groups

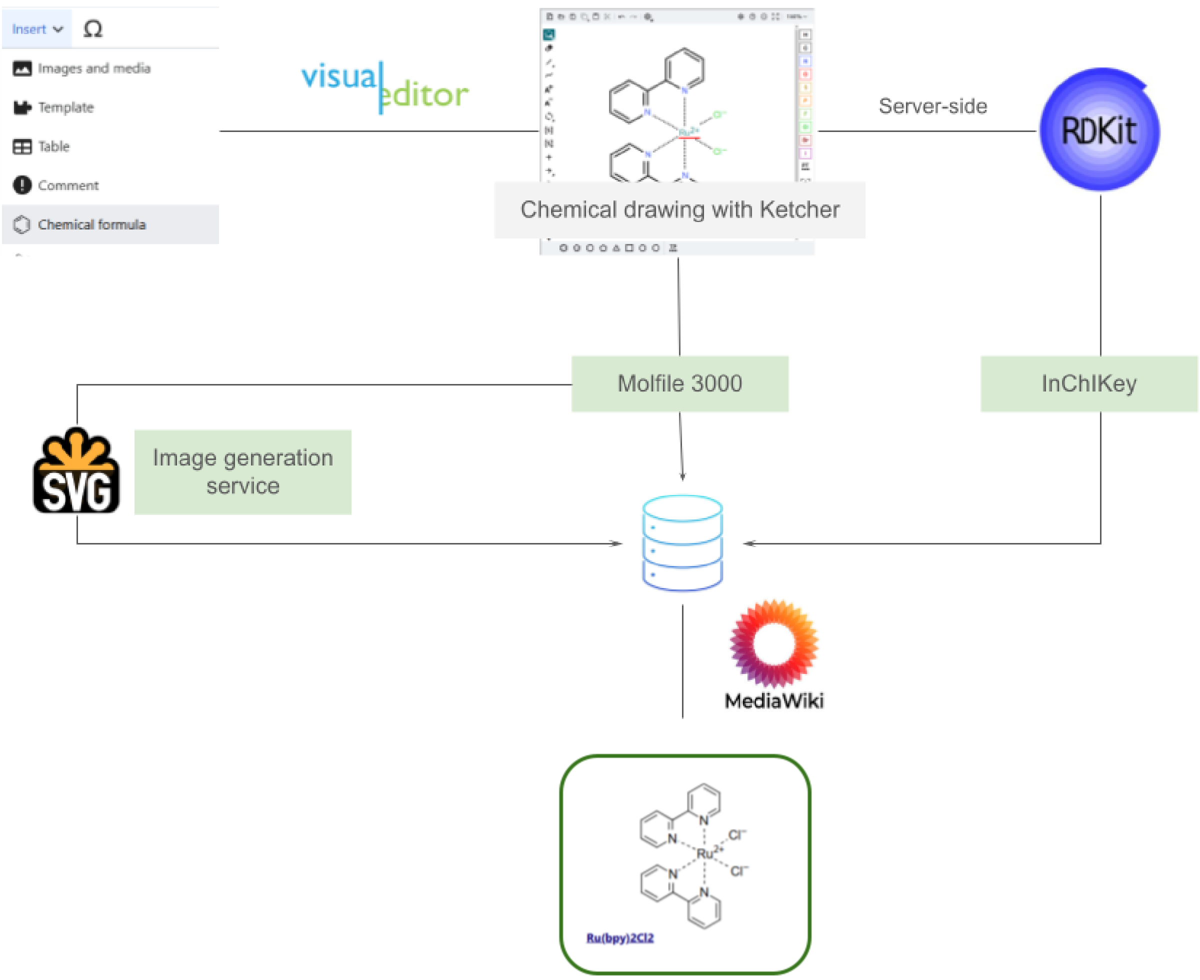

Figure S4. Schematic description of the data flow resulting in the generation and visualisation of a molecule when users enter a chemical formula in ChemWiki. The resulting image is annotated with either a unique number given for the molecule in the database or a name (shown here). The name can be assigned to the molecule in a separate action (not shown here).

For the generation of molecule groups, e.i. the generation of different molecules with the same core structure and different residues, an adapted workflow has to be followed: the generation

of a molecular group needs an additional step after the drawing of a chemical structure (with Ketcher editor) as the inserted R-groups need to be defined and parsed into the concrete molecules resulting from core and residues (Figure S5).

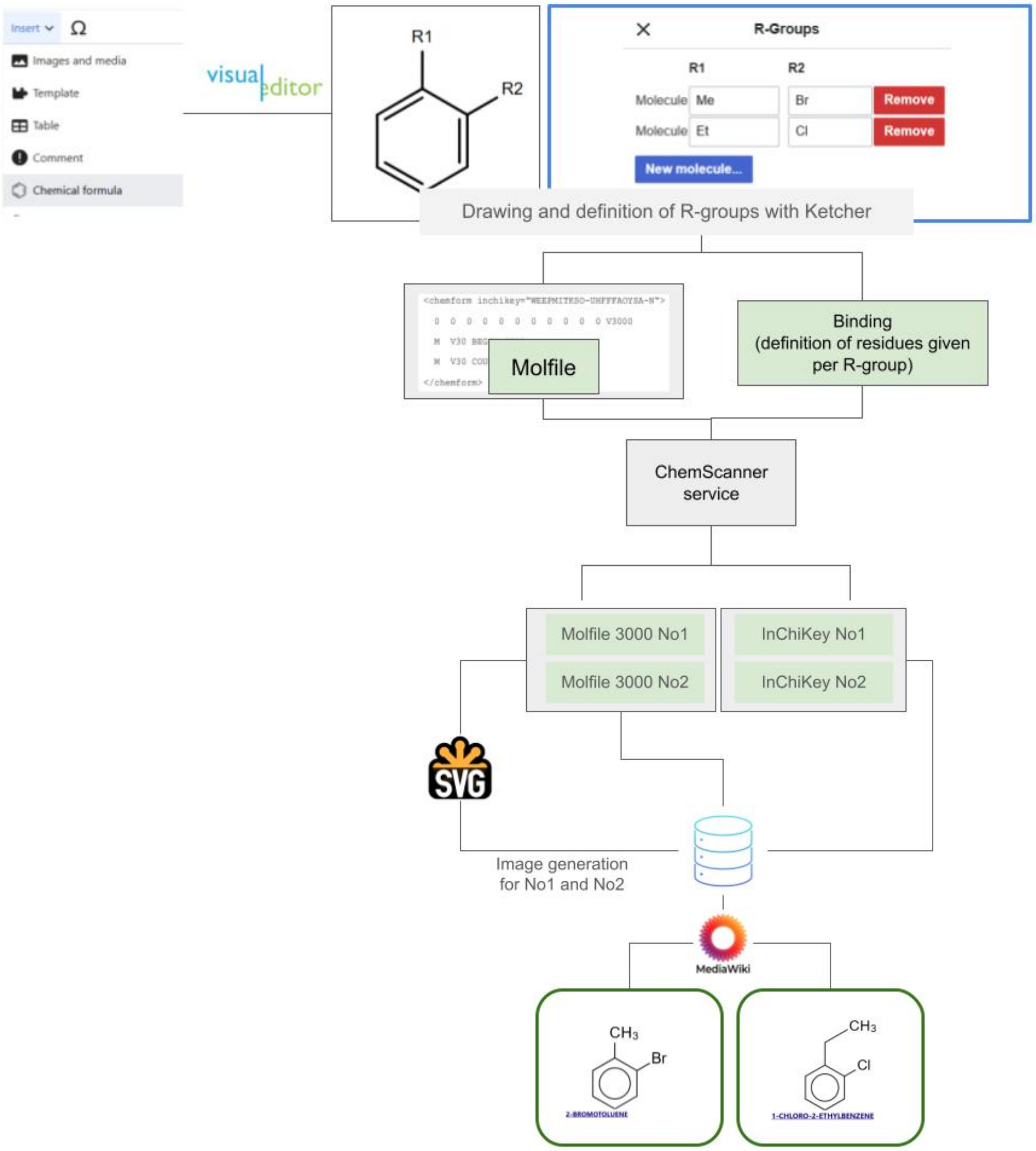

Figure S5. Data flow when users enter R-Groups for a molecule collection in ChemWiki.

8. Details for the Molecule Page

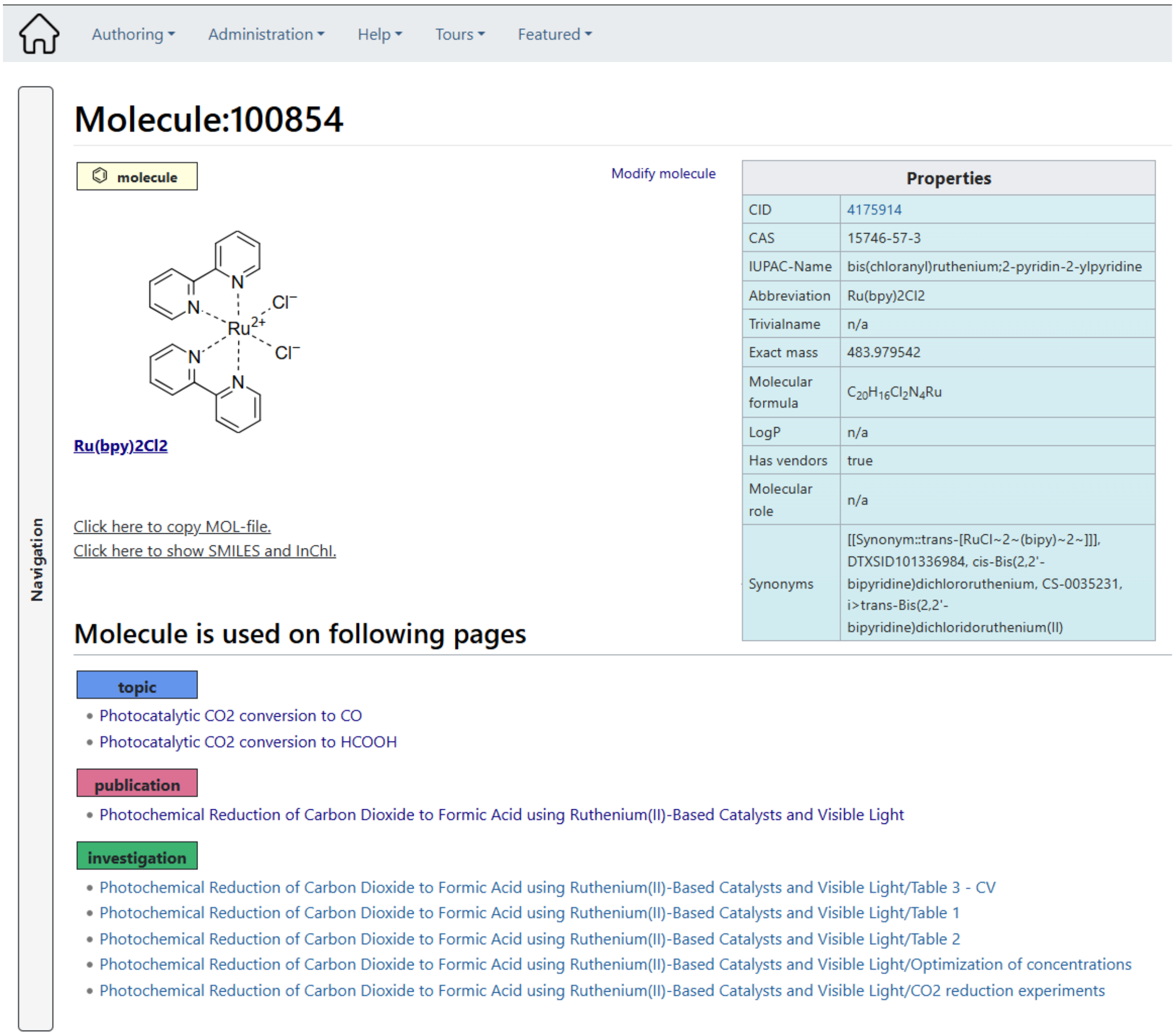

Figure S6. Screenshot of a typical Molecule Page (cut for representation purposes). The molecule can be edited in different ways: A modification of the molecular structure can only be done by users with special admin rights in the Wiki, a completion of the names and abbreviations can be done by every user in the edit mode of the page (not shown here).

9. Authors page

One of the featured pages of the Wiki is the authors page. The authors page lists all authors of publications in the Wiki, for that a publication Page (containing Literature) is described. Whenever available, authors are identified with their ORCID.

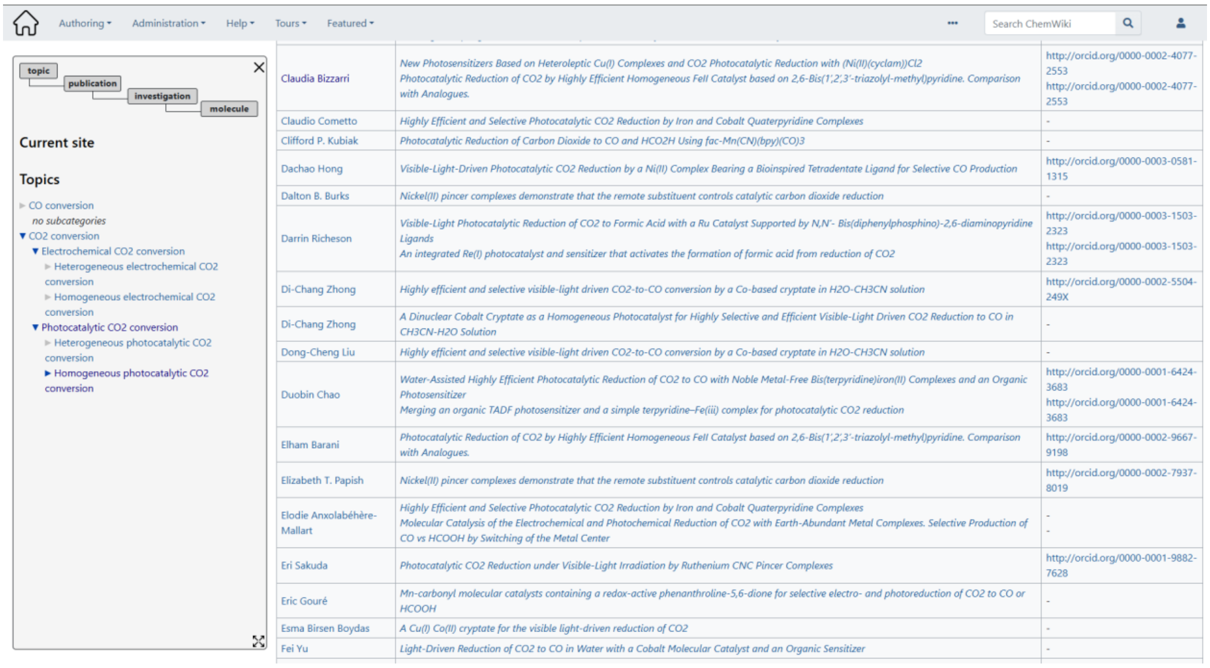

Figure S7. A screenshot of the authors' page with a summary of the publications assigned to the authors in the wiki and their ORCID.

10. Information on Ontologies and Terms used in the CKB Model

By using ontologies, concepts in a data model are assigned to terms in a controlled vocabulary. This has the benefit that knowledge encapsulated by the data is represented in a way that is structured and coherent across systems. This facilitates machine readability, re-use of data, and integration of different data sources. Describing data using ontologies furthermore enables faceted querying of the data.

Investigation: Definition from OBI: a planned process that consists of parts: planning, study design execution, documentation and which produce conclusion(s). A broad term denoting any investigation or experiment that yields data that are presented in a publication, including chemical reactions and physical measurements. There are two types, *Assay* (e.g. cyclic voltammetry measurement) and *Molecular Process* (e.g. photocatalytic CO_2 conversion). One individual row of an investigation table is called an *experiment.*

Experiment: A measurement or reaction done under specific specified conditions. If performed multiple times under slightly varied conditions, several *experiments* are compiled into one *investigation.*

Assay: Definition from OBI: A planned process with the objective to produce information about the material entity that is the evaluant, by physically examining it or its proxies. Measurement of physical data of a chemical substance under specific experimental conditions.

Molecular Process: Definition from MOP: A process in which at least one of the participants is a molecule.

Reactions between chemical substances (*molecules*) to products under specific experimental conditions.

Semantic MediaWiki allows the integration of external ontologies by assigning a special property *imported from.(Special property "Imported from")* We specifically utilize the ontologies OBI, CHEBI, and MOP to define classes in our data model, as indicated in Figure 2 by the dashed lines connecting to the LOD Cloud. As a result, data in our wiki, when retrieved in a linked data format such as RDF, is immediately compatible with other systems aware of the ontologies used.

11.Main limitations and opportunities of the CKB

Due to the technical implementation of the Wiki in its current form, limitations are given referring to the automatic update options:

- Currently, external data such as information from Pubchem or DataCite is imported and not requested live due to performance reasons. That means, the wiki does not profit from updates of external sources automatically and updates from external data sources would require a re-import.

General limitations resulting from Semantic MediaWiki as a technology stack:

- The wiki data model is in principle extendible and adaptable by the user, but it requires a deeper understanding of the wiki functionality and principles. A trained user would

be required for this task. For instance, adding new investigation types would require creating at least a new data input form as well as new rendering templates.

- Limitations of SMW's datamodel: SMW implements a knowledge graph that represents a discrete datamodel, i.e. it can represent only true facts, e.g. vagueness or probabilities needs cannot be expressed natively. Since there is a duality of the datamodel on the one hand and the means for data input (forms) and data presentation (templates and queries) extending and modifying the data model is not an easy task. In scientific domains where new discoveries might lead to new aspects that we would want to represent in CKB, this would also mean that the data model and its user-facing counter parts need to be updated.

Limitations and challenges referring to the maintenance of the Mediawiki Content

- The MediaWiki requires the maintenance by an active user community to claim comprehensive information about the depicted scientific topics and to ensure the correctness of the data. Therefore, the wiki needs to reach a certain level of acceptance in the community and the availability of skilled users who are intrinsically motivated to support the provision and curation of content.

The use of MediaWiki as a platform in combination with the implemented routines offer the following opportunities:

- The Wiki is based on standard technology with a large user community and freely available via GPL. That facilitates the maintenance of the system and its further extension. Technical updates to the system need to be added to the software from time to time, but due to the use of the well-known semantic mediawiki, these updates can include patches and upgrades (security aspects, functionality) that come for free.
- As mediawiki itself is a technology that is also well-known to users, the maintenance in terms of content related extension and curation can be done without extensive onboarding. This can help to solve the challenge of involving the community into the project.
- The wiki provides technology to organize scientific information in a way that is well-known to scientists. It allows to aggregate results of publications by creating overview pages (topcis) from a set of publications, allowing the automated summary of scientific results which is a huge effort without that support.
- These aggregation pages re-use data from publications pages that do not need to be rewritten or extracted. This avoids errors, saves a lot of time and effort and allows the

generation of summaries without time delay. The Wiki further augments data from external sources with manually curated data from the users, allowing a combination of information from different resources.

- The Wiki supports the combination of published content with access to machine-readable data (via Query or JSON-LD Export). Currently, the data comes mainly from (1) Investigations, (2) Molecules used and described in investigations, and (3) Annotations in publications. The availability of machine readable information is a big advantage in terms of supporting AI in the long run.
- The Wiki data model is extensible, also on user-level. The supported workflows in the wiki can be further enhanced, e.g. in a way that personal wiki spaces can be used for working on publications before publishing them.

Computational costs:

- Parts of the wiki content (eg. on topic pages) are aggregated from the semantic data model. This is logically a graph database, based on a conventional RDBMS (Relational Database Management System). For performance reasons, the rendered output is cached and only computed from time to time (also on demand if necessary).
- Other complex tasks concern rendering, creation, and transformation of molecules. This is provided by external services outside the wiki, not by the wiki itself and rely on common tools used for cheminformatic purposes (such as RDKit). Those tools certainly have their limitations but are used within a variety of cheminformatics services and are therefore established tools also for large user communities. Operations including automatic creation of new wiki pages are mostly done asynchronously (eg. when importing molecules from PubChem).
- With a growing number of users and page creations, the infrastructure and resources might need to be changed and extended. Therefore, an infrastructure that can be dynamically extended, is needed.

12. Possible further extensions with respect to the integration of cheminformatics tools and LLMs

In future enhancements to ChemWiki, the integration of additional Cheminformactics software as well as Large Language Models (LLMs) may play an important role in improving how chemical knowledge is formalized and accessed.

Use cases for Cheminformatics software

(1) Currently, the content of the CKB needs to be extracted from the literature and added to CKB manually. In the future, this task could be facilitated by Chemical Tagger (Hawizy et al. 2011) which allows to extract chemical entities and their relationships making the results available in a machine-processable format that could be used as input for the CKB.

(2) The Cheminformatics Software OPSIN(Lowe et al. 2011) could be used to convert chemical names (IUPAC names) into machine readable formats to be used to store the corresponding identifiers such as InChI and SMILES as well as molfiles in the CKB.

(3) The use of ChemScanner(Nguyen et al. 2019) could allow the recognition of chemical entities from chemical drawings embedded to manuscripts. This could support the identification of molecules but also reactions and their retrieval in machine usable manner to enrich the CKB.

Use cases for LLMs

(1) Given that LLMs have shown robust capabilities for knowledge extraction across various domains[3], we anticipate significant benefits for ChemWiki from their application. Such as to transform unstructured data from scientific publications into the structured formats used within publication pages, investigations, and descriptions of molecules. These models could extract experimental details, such as molecules, temperatures, and other parameters directly from textual sources in an "assistant" mode allowing to review and adjust the extracted values. (2)

Upon the importation of new publications into ChemWiki, LLMs could be used to automatically craft summaries that encapsulate the core findings, methodologies, and conclusions of the research. This enhances user engagement by allowing for quick scanning of articles to identify pertinent research.

(3) LLMs could also improve the query interface to make it more intuitive. Currently, users need to have a specific understanding of the data model to efficiently use the query interface. The proposed enhancement involves a natural language query interface that allows users to interact with the system in everyday language, significantly lowering the barrier to accessing complex data.

(4) The integration of a "Data Interpreter" could further automate and accelerate the expansion of the platform. This tool could dynamically generate a comprehensive plan for each new topic proposed by users, guiding the data ingestion process from literature search to the final publication on the site. The Data Interpreter could automate the search and selection of relevant papers, the creation of the structured data model in ChemWiki, and the generation of draft content for publication pages.

13. Software dependencies

Software	License	Details
Ubuntu Linux	GPLv2	https://ubuntu.com/legal/intellectual-property-policy
Apache Webserver	Apache 2.0	
MariaDB	GPLv2	
Apache SOLR (Wiki-Suche)	Apache 2.0	
Mediawiki	GPLv2	
ChemExtension	GPLv2	
WikiFarm	GPLv2	

extensions: SMW, PageForms	GPLv2	
Ketcher	Apache 2.0 License	Version 2.5.0
Indigo toolkit	Apache 2.0 License	Version 1.7.1.0
ChemScanner	GNU AGPLv3 License	
Chemscanner-api		https://github.com/CamAnNguyen/chemscanner_api
Molecule rendering by Indigo service	Apache 2.0 License	Version 1.28.0

1. [No title]. https://terminology.tib.eu/ts/.
2. *doc/ChemExtension.md at Main · ComPlat/ChemistryKnowledgeBase*. (Github).
3. Xu, D. *et al.* Large language models for generative information extraction: a survey. *Front. Comput. Sci.* **18**, (2024).
4. Nechakhin, V., D'Souza, J. & Eger, S. Evaluating Large Language Models for structured science summarization in the Open Research Knowledge Graph. *Information (Basel)* **15**, 328 (2024).
5. Martinez, F. *et al.* Study-buddy: A knowledge graph-powered learning companion for school students. in *Lecture Notes in Computer Science* 133–137 (Springer Nature Switzerland, Cham, 2023). doi:10.1007/978-3-031-43458-7_25.
